# Phylogenetic Characterization of the Palyam Serogroup Orbiviruses

**DOI:** 10.3390/v11050446

**Published:** 2019-05-16

**Authors:** Karen Ebersohn, Peter Coetzee, Louwrens P. Snyman, Robert Swanepoel, Estelle H. Venter

**Affiliations:** 1 Department of Veterinary Tropical Diseases, Faculty of Veterinary Science, University of Pretoria, Onderstepoort 0110, South Africa; lokisnyman@gmail.com (L.P.S.); bob.swanepoel@up.ac.za (R.S.); estelle.venter@jcu.edu.au (E.H.V.); 2 Equine Research Centre, Faculty of Veterinary Science, University of Pretoria, Onderstepoort 0110, South Africa; peter.coetzee@yahoo.com; 3 College of Public Health, Medical and Veterinary Sciences, Discipline Veterinary Science, James Cook University, Townsville 4811, Australia

**Keywords:** Palyam, sequencing, phylogenetic analysis

## Abstract

The Palyam serogroup orbiviruses are associated with abortion and teratogenesis in cattle and other ruminants. Of the 13 different serotypes that have been identified, the full genome sequence of only one, Kasba, has been published. We undertook to perform Next Generation Sequencing (NGS) and phylogenetic analysis on 12 Palyam serotypes plus field isolates of the African serotypes in our possession. The Palyam serogroup was found to be most closely related to the African horse sickness virus group and showed the most distant evolutionary relationship to the equine encephalosis viruses (EEV). Amino acid sequence analysis revealed that the gene encoding VP7 was the most conserved within serotypes and VP2 and VP5 showed the highest degree of variation. A high degree of sequence identity was found for isolates from the same geographical region. The phylogenetic analysis revealed two clades where the African serotypes were all very closely related in one clade and the other clade contained the Australian and Asian serotypes and one African serotype, Petevo. It was evident from the sequence data that the geographical origin of Palyam serogroup viruses played an important role in the development of the different serotypes.

## 1. Introduction

The Palyam serogroup of the genus *Orbivirus* and family *Reoviridae* are arthropod-borne viruses that have been isolated in Asia, Africa, and Australia [1,2]. Some of the viruses are associated with abortion and teratogenesis in cattle and other ruminants [3,4,5,6]. Currently, 13 serotypes are recognized by the International Committee on Taxonomy of Viruses (ICTV): Palyam, Kasba, Vellore, Abadina, D’Aguilar, Nyabira, CSIRO Village, Marrakai, Gweru, Bunyip Creek, Petevo, Marondera and Kindia. The serotypes are defined by the serum neutralisation test, but cross-reactions occur, separating the viruses into six antigenic complexes [1,7]. Although some Palyam serogroup viruses had been isolated previously from aborted foetuses [7,8], it was only after an outbreak of congenital abnormalities in cattle in Japan in 1985–1986 that their pathogenic importance was investigated [6,9].

The Palyam viral genome is similar to that of other orbiviruses, which notably include viruses like bluetongue virus (BTV), epizootic haemorrhagic disease virus (EHDV), and African horse sickness virus (AHSV). The genome consists of ten linear double-stranded (ds) RNA segments that code for seven structural viral proteins (VP1 to VP7) and four non-structural proteins (NS1, NS2, NS3 and NS3a) [7,10,11]. Non-structural protein (NS4) has been detected for other orbiviruses, such as BTV and AHSV [12,13], but has not been described for Palyam viruses. Segments 1, 2, 3, 4, 6, 7 and 9 of the Palyam genome encode for VP1, VP2, VP3, VP4, VP5, VP7 and VP6, respectively [10]. Segment 5 encodes for NS1 and Segment 8 for NS2. NS3 and NS3a are encoded by Segment 10, which is also the smallest gene [14].

Sequence data are lacking for most of the Palyam serogroup, with Kasba being the only serotype for which complete genome sequences are available [10,15,16]. In addition, sequences are available for certain genome segments of the serotypes from Japan, Australia and Zimbabwe [17]. Complete sequence data for all serotypes would confirm the validity of the taxa defined by serology and give better insight into the genetic heterogeneity of serotypes and their relationship to other orbiviruses. The data would facilitate determining the source of viruses in outbreaks, allow for monitoring of re-assortment and recombination between viruses, and enable the development of molecular diagnostic assays. Hence, the aim of the present study was to sequence the full genomes of members of the serogroup in our possession. We did not have access to the Kindia virus [18].

## 2. Materials and Methods

All viruses used in the study were obtained from the National Institute for Communicable Diseases (NICD), Johannesburg, South Africa. The prototype Palyam serogroup viruses, except for Apies River, Gweru, Marondera and Nyabira, were originally obtained from the Yale Arbovirus Research Unit, New Haven, Connecticut, USA, and propagated, freeze-dried and stored at NICD. All Nyabira, Gweru and Marondera isolates originated from Zimbabwe and the Apies River isolate from Gauteng Province, South Africa [7]. The viruses had been isolated from various sources at the times and locations indicated in Table 1. Viruses were propagated and passaged once in Vero (African green monkey kidney) cells (ATCC, Manassas, VA, USA, CRL #1587), using Eagles medium (Biochrom^®^, Berlin, Germany) containing 5% foetal bovine serum (Gibco^®^, ThermoFisher Scientific, Waltham, MA, USA) and 1 mg/mL gentamycin (Virbac^®^, Centurion, South Africa).

Total RNA was extracted from cell culture material using Trizol™ (Invitrogen ThermoFisher Scientific, Waltham, MA, USA), in accordance with the manufacturer’s instructions. Single-stranded RNA (ssRNA) was removed by adding 2M LiCl to the samples as previously described [19]. The dsRNA was purified using the MinElute Gel Extraction Kit (QIAGEN^®^, Hilden, Germany)) in accordance with the manufacturer’s instructions. Full-length amplification of cDNA (FLAC) was performed as previously prescribed [19,20]. The DNA amplicons obtained during the FLAC assay were sent to a commercial service provider, Inqaba Biotech^®^ (Pretoria, Gauteng, South Africa) for sequencing. The amplicons were sequenced on an Illumina^®^ Mi-Seq sequencer (Illumina, Inc, San Diego, CA, USA), using the Nextera XT DNA sample preparation kit (Illumina, Inc, San Diego, CA, USA) and 300-bp paired-end V3 Illumina chemistry.

Sequence data generated by Illumina sequencing were analysed using the CLC Genomics Main workbench version 8.0.1 (http://www.clcbio.com/). De novo assembly of sequence reads was performed and contig sequences were prepared. The contigs were aligned with sequences on the National Centre for Biotechnology (NCBI, http://www.ncbi.nlm.nih.gov/) database by the Basic Local Alignment Search Tool (BLAST) (https://blast.ncbi.nlm.nih.gov/Blast.cgi) for verification of the different gene sequences. The reads were mapped using the contigs as reference sequences. Where sequences were mapped to a reference sequence, a consensus was extracted. Translation was done for all of the sequences to ensure that open reading frames (ORF) were maintained.

The sequences for the 10 genes of all the serotypes were aligned separately, together with an out-group, AHSV. Concatenated genes were also constructed for all the serotypes, including the field isolates as well as four different orbiviruses: AHSV, BTV, EEV and EHDV. The sequences for the other orbiviruses were obtained from GenBank (NCBI nucleotide database, https://www.ncbi.nlm.nih.gov/genbank/). The accession numbers of all the new sequences that were obtained, as well as other sequences used for phylogenetic analysis, are listed in Table 2. Sequences were aligned and pairwise comparisons created for each gene using the CLC Genomics Main workbench. The distance between the sequences was calculated for each gene as well as the percentage sequence identities. The ORF’s were identified for the different gene sequences of all the serotypes and the sequences were translated into proteins. The protein sequences were then aligned and pairwise comparisons generated for each gene, using the CLC Genomics Main workbench version 8.0.1 (http://www.clcbio.com/). The distance between sequences was calculated for each gene as well as the percentage sequence identities.

All sequences were aligned using the online version of MAFFT (https://mafft.cbrc.jp/alignment/server/) using default parameters. The aligned matrix was viewed, edited and truncated using MEGA X (www.megasoftware.net) [21]. The alignments were converted into nexus and phylips files using the online Format Converter Tool (www.hiv.lanl.gov). Data-display networks (DDN) (neighbour-networks) were constructed with SplitsTree 4 [22]. The support values calculated were based on 1000 bootstrap replicates and the networks were based on uncorrected p-distances using all characters.

The phylips files were used to initiate model estimation via jModel test2 [23], using the online portal Cipres Science gateway [24] where both AIC (Akaike information criterion) and BIC (Bayesian information criterion) criteria were consulted for analysis. When AIC and BIC differed in model selection, AIC estimation was favoured. Bayesian analysis was performed in MrBayes (MrB) version 3 [25]. The data for the concatenated sequences were partitioned into gene regions, unlinked with previously estimated models assigned to the appropriate partitions. The probability density rates were set to a flat dirichlet and using the Markov Chain Monte Carlo (MCMC) method, four chains were run simultaneously for 1 million generations. Trees were sampled and saved for every 500th generation and 10% of the trees sampled were discarded as burn-in. Posterior probabilities were calculated from the remaining saved majority-rule consensus trees and a value of 0.95 was viewed as statistically significant. Tracer v1.6 (http://beast.bio.ed.ac.uk/Tracer) was used to investigate the effective sample size (ESS) of the Bayesian analysis, where values >200 were viewed as an appropriate sample size. RAxML, version 8.2.4 [26] was employed for a maximum likelihood (ML) analysis of the same partitioned dataset. GTR (Generalized time-reversible) models were used for analysis including either gamma distribution, or invariable sites, or both, as estimated by the jModel Test. The autoMRE function was invoked for bootstrap calculations. Bootstrap values from the ML analysis were superimposed on the phylogeny recovered from the Bayesian analysis.

## 3. Results

### 3.1. Nucleotide Sequences

Complete sequences were obtained for all the genes of the viruses submitted, except for Segment 1 of Vellore and Nyabira 1772. The Segment 1 sequence for Vellore had a gap of 46 bp, from position 2351 to 2397, from the total of 3930 bp, in regard to the reference sequence. The sequence for Nyabira 1772 had three large sections in the sequence, with only 2088 bp available of the total 3930 bp. Sufficient quantities of the original samples were not available to repeat the sequencing or to design primers to complete the entire region with conventional sequencing. All of the Segment 1 sequences except for Nyabira 1772 were submitted to GenBank (NCBI nucleotide database, https://www.ncbi.nlm.nih.gov/genbank/) and the accession numbers are given in Table 2. The partial Segment 1 sequence of Nyabira 1772 was included in the dataset for phylogenetic analysis.

Analysis of the nucleotide sequence identity of the concatenated dataset indicated that the identity ranged between 45.91% and 53.52% when comparing Palyam viruses with the other orbiviruses and between 76.24% and 99.84% within the Palyam serogroup. AHSV was the orbivirus that was the most closely related to the Palyam viruses and had the highest sequence identity value when compared to the Palyam viruses (53.07%–53.52%). EEV had the lowest sequence identity (46.27%–46.56%) and the biggest genetic distance when compared with the Palyam viruses.

The nucleotide sequence identity for the concatenated dataset of the Palyam serogroup serotypes and field isolates ranged between 77.14% and 97.22%. The percentage sequence identity for the Gweru prototype and Gweru field isolates ranged between 99.41% and 99.79%, showing that these isolates are closely related. The sequence identity between the Nyabira prototype and the Nyabira field isolate was 96.38%. The range for the Abadina samples was between 99.77% and 99.78%.

### 3.2. Amino Acid Sequences

The amino acid sequence identities ranged between 77.23% and 100% for all the genome segments of the Palyam serogroup serotypes with the exception of Segment 2. This segment, coding for VP2, showed the greatest variation, with a percentage sequence identity ranging between 35.26% and 99.9% (Table 3). The viruses with the most similar sequences for VP2 were Gweru and CSIRO Village (99.9%), Marondera and Apies River (98.72%) and Vellore and Kasba (98.11%). Palyam and Petevo showed the biggest variation in sequence identity (35.26%).

VP5 (Segment 6) exhibited the second most variable range of sequence identity (77.23–100%) (Table S3) Vellore and Kasba had identical sequences for VP5, as did Marondera and Apies River viruses. The percentage sequence identity for VP6 (Segment 9) ranged between 85.3% and 100%, with Vellore and Kasba displaying the same amino acid sequence (Table S4). The range was from 89.1% to 100% for NS2 (Segment 8), with Gweru and Nyabira being identical. 

The amino acid sequences for VP7 were the most conserved of all the proteins across the serogroup, with percentage sequence identity ranging between 97.75% and 100% (Table 3). The sequences for Abadina, Nyabira, Apies River and Marondera were identical for VP7. Bunyip Creek, D’Aguilar and CSIRO Village also had identical VP7 sequences. The segment coding for VP3 was also highly conserved (96.81–100 % sequence identity). Gweru, Nyabira and Marondera had the same amino acid sequences for VP3 (Table S2).

The percentage sequence identity for VP1 ranged from 92.71% to 99.54% (Table S2) and the range for NS1 was between 89.1% and 100% (Table S4). Apies River, Marondera and Nyabira had the same amino acid sequence for NS1. VP4 (Segment 4) had a sequence identity ranging from 92.08% to 99.53% (Table S3) and NS3 (Segment 10) had a range between 91.28% and 100% (Table S5). Identical NS3 sequences were identified for Gweru, Nyabira and Abadina. Several ORF’s in both directions were detected in Segment 9 and further analysis needs to be done to investigate the presence of a sequence coding for NS4.

### 3.3. Phylogenetic Trees

**Figure 1:**
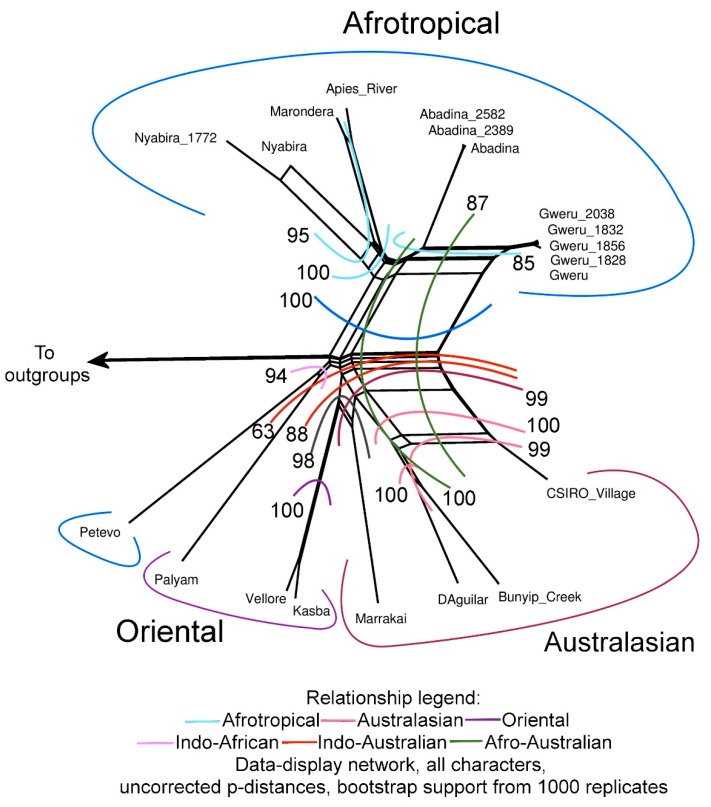
Data-display network for all the serotypes of the Palyam serogroup used in this study. Different coloured lines present bootstrap support for different intra- and inter-geographical relationships among serogroups. Arrow to outgroups: AHSV, BTV, EEV and EHDV.

**Figure 2:**
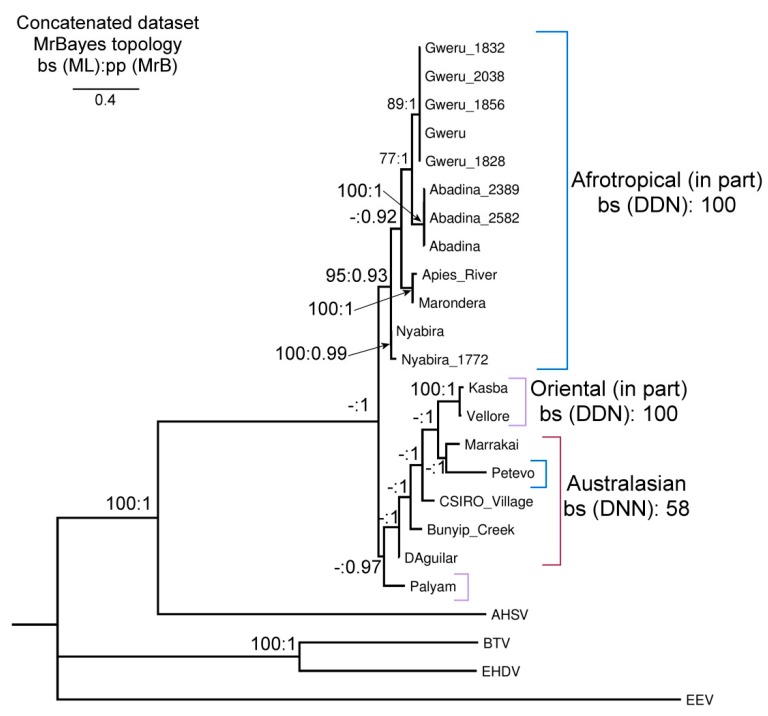
Phylogenetic tree for the concatenated dataset of the Palyam serogroup of orbiviruses used in this study. Bootstrap values from the Maximum Likelihood analysis were superimposed on the posterior probabilities gained from Bayesian analysis (bs: pp), bs values <75 and pp values <0.9 are not displayed and are represented by a “-“.

The final concatenated sequence matrix comprised 20 in-group taxa, four out-group taxa (AHSV, BTV, EHDV & EEV) and 20775 nucleotides from 10 genes. Similar topologies were recovered from the Splitstree (DDN) and Bayesian (MrB) analysis (Figure 1 and Figure 2), both with higher support in comparison to the ML analysis. The phylogenetic tree constructed from the concatenated dataset (Figure 2) showed a common ancestor between the Palyam serogroup viruses and AHSV. When looking at the other orbiviruses used as out-groups in this study, it was evident that the orbiviruses all shared a common ancestor, but that AHSV was the most closely related to the Palyam serogroup viruses and EEV most distantly related. The Palyam serogroup viruses formed a monophyletic clade with strong support from the MrB analysis (pp: 1), with the out-groups in the DDN separated as a clade with a bootstrap value of 100.

The Afrotropical serotypes, excluding Petevo, formed a well-supported monophyletic clade (Figure 1 and Figure 2, DDN: 100, MrB: 1). Within the clade, Apies River and Marondera were recovered as closely related sister taxa (DDN: 100, ML: 100, MrB: 1). Nyabira was recovered as either sister to Apies River and Marondera (DDN: 100), or sister to the remaining Afrotropical taxa (ML: 95, MrB: 0.93). Abadina and Gweru represent the most derived sister relationship (DDN: 85, ML: 77, MrB: 1). Together the Oriental and Australasian serotypes formed a second monophyletic clade (DDN: 63, MrB: 1). Petevo, an Afrotropical serotype fell within this clade in both the ML and Bayesian analysis. A monophyletic Australasian clade was recovered with the DDN (bs: 99), in contrast to the topologies recovered from the ML and Bayesian analysis where Kasba, Vellore and Petevo fell within the Australasian group. Kasba and Vellore are closely related sister taxa with strong support across all methods (DDN: 100, ML: 100: MrB: 1). Marrakai seems to be a probable sister group to Kasba and Vellore (DDN: 98, MrB: 1), however, Marrakai was recovered as a sister to the Petevo virus in the phylogenetic analysis, which was not supported by the DDN.

The placement of the Petevo and Palyam viruses remain uncertain with the two isolates forming a sister group in the DDN (bs: 94). Petevo fell within the Oriental and Australasian clade in the MBayes topology, whereas Palyam was a sister to the Oriental and Australasian clade in the DDN. From the DDN, CSIRO Village shared some similarities with the Afrotropical serotypes, with clade support with Gweru (bs: 87) and Gweru and Abadina (bs: 100).

## 4. Discussion

In this study NGS was used to obtain full genome sequences for 12 of the 13 recognized serotypes of the Palyam serogroup. The newly generated sequences allowed for a more complete phylogenetic analysis of the different serotypes and their relationship to other orbiviruses. The minimum and maximum percentage identities for the nucleotide and amino acid sequences between and within serotypes were determined as well as the relationship of Palyam viruses to other orbiviruses. Sequences for field isolates of the Gweru, Abadina and Nyabira serotypes were also obtained and included in the phylogenetic analysis.

Analysis of the nucleotide sequence identity of the concatenated dataset indicated that AHSV was the orbivirus that was the most closely related to the Palyam viruses and had the highest sequence identity value when compared to the Palyam viruses. EEV had the lowest sequence identity and the biggest genetic distance when compared with the Palyam viruses.

During analysis of the amino acid sequences of the separate genes of the Palyam serogroup serotypes, VP7 was found to be the most conserved of the genes with a sequence identity of between 97.75% and 100%, with Abadina, Nyabira, Apies River and Marondera having identical sequences. These viruses were all from the African region. The sequences for Bunyip Creek, D’Aguilar and CSIRO Village, viruses from Australia, were also the same but defined a second distinguishable group. The highly conserved VP7 would make a suitable target for a group-specific assay for the detection of Palyam viruses. VP3 was also highly conserved across the serogroup. Sequence identities ranged from 96.81% to 100%, with indistinguishable sequences for Afrotropical serotypes Gweru, Nyabira and Marondera. VP7 and VP3 are major structural proteins which form the inner capsid and are involved with molecular interactions during virus assembly, therefore conserved [10,15]. This is similar to sequence data obtained from other studies on segment 7 of the Palyam serogroup viruses from Japan, Australia and Zimbabwe [15,16,27], where sequence identities of 97.3%–100% were observed for Kasba (Chuzan), D’Aguilar, Marrakai, CSIRO Village, Marondera, Gweru and Nyabira viruses. Sequence data for other orbiviruses such as BTV has also demonstrated that VP7 and VP3 are more highly conserved between serotypes than the outer capsid proteins, VP2 and VP5 [28].

The amino acid sequences for VP2 and VP5 showed the highest degree of variation, with VP2 being the more variable of the two. These proteins form the outer capsid of the virus and are responsible for viral neutralisation and serotype specificity, hence the variability of the sequences between the serotypes. Studies on BTV have obtained similar results, where it was found that segments coding for VP2 and VP5 are the most variable segments of the genome [29].

Phylogenetic analysis indicated that the Palyam serogroup viruses were the most closely related to, and shared a common ancestor with, AHSV. BTV and EHDV form a sister group and EEV shows the most distant evolutionary relationship to the Palyam serogroup viruses, as has been found previously [3].

The phylogenetic analysis revealed two well-supported and distinct clades, one containing all Afrotropical strains (except for Petevo), and one clade composed of Petevo plus all of the strains from the Orient and Australasian regions. The overall topology of the Bayesian analysis had stronger support throughout, compared to the phylogram inferred from the ML analysis. In RA x ML, only incomplete model partitioning between genes, using GTR-based models is possible. The selected models and subsequent data partitioning had a significant effect on the data analysis, and Bayesian analysis with partitioning similar to that of RA x ML produced weaker trees.

The African serotypes, contained in one clade, are all closely related, with identical sequences for several gene segments, namely segments 3, 5, 7, 8 and 10 (Table S2, Table S4, Table S5 and Table 3). Apies River and Marondera are the most closely related, with a 97.22% sequence identity, the sequences were identical for VP5, VP7 and NS1 and the identity was >96.42% for all other segments. This similarity is comparable to the genetic variability within the two Nyabira isolates (96.38%), suggesting a revision of the proposed status of Apies River virus as a separate serotype [30]. The serotypes of African origin are distinct from serotypes of Australian and Asian origin, with the exception of Petevo virus (Figure 1 and Figure 2).

The second clade contained the Australian and Asian serotypes. Palyam was the first virus to be isolated in 1956 in India and fell as the out-group to the remaining Australasian and Oriental taxa, indicating an ancestral status. The Marrakai serotype seems to form a sister group to both the remaining Australian serotypes and the oriental Vellore and Kasba, which might be indicative of recombination between previously geographically-isolated serotypes (Figure 2). This is concurring with findings in previous RNA hybridization studies, where the data indicated that the Australasian gene pool is defined by three of the four Australian serotypes [31] and with phylogenetic analysis of segment 7 of Palyam viruses [15]. When comparing the Australian viruses, the sequence identities for CSIRO Village, D’Aguilar and Bunyip Creek were high but Marrakai had lower-sequence identity values in this comparison. The Marrakai virus was isolated from midges and is also the only Australian serotype that had not been isolated from cattle. The Marrakai virus forms a sister group with Petevo (Figure 2), the only African (Central African Republic) serotype to group in this clade this was well-supported by the Bayesian analysis. The Petevo virus was isolated from Ixodid ticks [32]. Interestingly, in the DDN, the Petevo virus and the Palyam virus had a strong sister relation (bs. 94). The unexpected placement of Petevo outside of the Afrotropical clade is possibly due to translocation of the virus via infected vectors or hosts to Africa from Asia or Australasia after the establishment and diversification of the remaining Afrotropical strains.

The Kasba and Vellore viruses also form sister groups and are very closely related (97.01% sequence identity). The high-sequence identity that exists between the viruses from Australia and Asia may suggest that there has been some gene-flow between the serotypes, possibly due to the cattle from Indian descent being introduced to Australia during cattle trade. The CSIRO Village serotype shares a curious genetic similarity with the Afrotropical Gweru and Abadina viruses (DDN: 87) and is worth investigating. The similarity could be due to intermitted gene-flow between the regions, possibly with the trade of infected hosts, or may reflect evolutionary convergence.

It is clear from the sequence data that the geographical origin of viruses of the Palyam serogroup played an important role in the development of the different serotypes. The clustering of the viruses into the regional groups reflect the evolution of the serotypes in the different areas of the world. Since similar vectors are found in both Australasia and the Afrotropics, the presence of closely related vector species in distant geographic regions could have allowed for the establishment and diversification of introduced viruses due to local episystem factors. A study comparing certain viruses from Japan, Australia and Zimbabwe [17] concluded that Palyam serogroup viruses evolved independently in separate gene pools, but that isolates from the same geographical area are closely related at nucleotide and amino acid levels, even if they are different serotypes. This was confirmed by findings in this study. The evolution of the different Palyam serotypes could be explained by the spread of the ancestral virus from India to Northern Africa and Australia during cattle trade. Subsequently, the Afrotropical viruses (excluding Petevo) diversified, whereas gene-flow between the Australasian and Orient seems more complex. The viruses of these two regions are very closely related and do not form a separate clade according to region of origin as the viruses from Africa do. It is probable that the continued trade of infected hosts, or long-distance wind-borne dispersal of infected vectors between Australia and Asia [33] resulted in intermitted gene-flow that, for example, may have resulted in the possible close relation between Marrakai and the Oriental Kasba and Vellore viruses.

When comparing the different serotypes within the Palyam serogroup viruses a high degree of sequence identity was found between isolates from the same geographical region. For the Abadina isolates the sequence identity was >99.7% and for Gweru >99.41%. The two Nyabira isolates were the most variable, with a value of 96.38%. However, insufficient sequence data on isolates of the same serotype is available and therefore more phylogenetic analysis needs to be done before re-assortment between and within serotypes can be investigated. The only known serotype which was not included in this study and for which no sequence data is available is Kindia.

## 5. Conclusions

The full genome sequences of the different serotypes of the Palyam serogroup viruses, as well as selected field isolates from Africa, were obtained. The phylogenetic analysis revealed two clades; the African serotypes were all closely related in one clade, with identical sequences for several gene segments. The second clade contained the Australian and Asian serotypes and one African serotype, Petevo. The high-percentage sequence identity (85.6%–77,5%) that exists between the viruses from Australia and Asia may suggest that there has been some gene-flow between the serotypes. It was clear from the sequence data that the geographical origin of Palyam serogroup viruses played an important role in the development of the different serotypes.

The sequence data generated during this study could enable further investigation into the molecular evolution of viruses within the Palyam group with regard to re-assortment, genetic drift and intragenic recombination. Divergence-time estimation analysis can be investigated and compared to the period of emergence of cattle trade for a more accurate picture of the biogeography of Palyam viruses.

## Figures and Tables

**Table 1:** Palyam virus isolates used in the study.

Serotype	Strain Designation	Country of Isolation	Source of Isolation	Year of Initial Isolation
Prototypes				
Abadina	Ib Ar 22388	Nigeria	*Culicoides* spp.	1967
Bunyip Creek	CSIRO 87	Australia	*Culicoides schultzei*	1976
CSIRO Village	CSIRO 11	Australia	*Culicoides* spp.	1974
D’Aguilar	B8112	Australia	*Culex brevitarsus*	1968
Kasba	GG15534	India	*Culex vishnui*	1957
Marrakai	CSIRO 82	Australia	*Culicoides* spp.	1975
Palyam	G5287	India	*Culex vishnui*	1956
Petevo	Ar TB 2032	Central African Republic	*Amblyomma variegatum*	1978
Vellore	68886	India	*Culex pseudovishnui*	1956
Apies River	O/4518	South Africa	Cow blood	2005
Gweru	VRL1726/76	Zimbabwe	Bovine foetus	1976
Marondera	VRL1070/78	Zimbabwe	Cow viscera	1978
Nyabira	VRL792/73	Zimbabwe	Bovine foetus	1973
Field isolates				
Abadina 2582	2582/78	Zimbabwe	Bovine foetus	1978
Abadina 2389	2389/76	Zimbabwe	Bovine foetus	1976
Nyabira 1772	1772/74	Zimbabwe	Bovine foetus	1974
Gweru 1828	1828/82	Zimbabwe	Bovine foetus	1982
Gweru 1832	1832/79	Zimbabwe	Bovine foetus	1979
Gweru 2038	2038/76	Zimbabwe	Bovine foetus	1976
Gweru 1856	1856/76	Zimbabwe	Bovine foetus	1976

**Table 2:** Accession numbers of Palyam virus sequences submitted to GenBank and other orbiviruses used in the analysis.

Virus isolate	Segment 1 (VP1)	Segment 2 (VP2)	Segment 3 (VP3)	Segment 4 (VP4)	Segment6 (VP5)	Segment 9 (VP6)	Segment 7 (VP7)	Segment5 (NS1)	Segment8 (NS2)	Segment 10 (NS3)
Abadina	MH782454	MH823477	MH817078	MH817097	MH817117	MH823377	MH823397	MH823417	MH823437	MH823457
Bunyip Creek	MH782455	MH823478	MH817079	MH817098	MH817118	MH823378	MH823398	MH823418	MH823438	MH823458
CSIRO Village	MH782456	MH823479	MH817080	MH817099	MH817119	MH823379	MH823399	MH823419	MH823439	MH823459
D’Aguilar	MH782457	MH823480	MH817081	MH817100	MH817120	MH823380	MH823400	MH823420	MH823440	MH823460
Kasba	MH782458	MH823481	MH817082	MH817101	MH817121	MH823381	MH823401	MH823421	MH823441	MH823461
Marrakai	MH782459	MH823482	MH817083	MH817102	MH817122	MH823382	MH823402	MH823422	MH823442	MH823462
Palyam	MH782460	MH823483	MH817084	MH817103	MH817123	MH823383	MH823403	MH823423	MH823443	MH823463
Petevo	MH782461	MH823484	MH817085	MH817104	MH817124	MH823384	MH823404	MH823424	MH823444	MH823464
Vellore	MH782462	MH823485	MH817086	MH817105	MH817125	MH823385	MH823405	MH823425	MH823445	MH823465
Apies River	MH782463	MH823486	MH817087	MH817106	MH817126	MH823386	MH823406	MH823426	MH823446	MH823466
Gweru	MH782464	MH823487	MH817088	MH817107	MH817127	MH823387	MH823407	MH823427	MH823447	MH823467
Marondera	MH782465	MH823488	MH817089	MH817108	MH817128	MH823388	MH823408	MH823428	MH823448	MH823468
Nyabira	MH782466	MH823489	MH817090	MH817109	MH817129	MH823389	MH823409	MH823429	MH823449	MH823469
Abadina 2582	MH782467	MH823490	MH817091	MH817110	MH817130	MH823390	MH823410	MH823430	MH823450	MH823470
Abadina 2389	MH782468	MH823491	MH817092	MH817111	MH817131	MH823391	MH823411	MH823431	MH823451	MH823471
Gweru 1828	MH782469	MH823492	MH817093	MH817112	MH817132	MH823392	MH823412	MH823432	MH823452	MH823472
Gweru 1832	MH782470	MH823493	MH817094	MH817113	MH817133	MH823393	MH823413	MH823433	MH823453	MH823473
Gweru 2038	MH782471	MH823494	MH817095	MH817114	MH817134	MH823394	MH823414	MH823434	MH823454	MH823474
Gweru 1856	MH782472	MH823495	MH817096	MH817115	MH817135	MH823395	MH823415	MH823435	MH823455	MH823475
Nyabira 1172	NA	MH823496	MK007563	MH817116	MH817136	MH823396	MH823416	MH823436	MH823456	MH823476
AHS 1 Isolate E160445	KX987198	KX987199	KX987200	KX987201	KX987202	KX987203	KX987204	KX987205	KX987206	KX987207
BTV Isolate BTV10 IND2003k3	KP339244	KP339245	KP339246	KP339247	KP339248	KP339249	KP339250	KP339251	KP339252	KP339253
EEV Isolate Kimron1	AB811635	AB811636	AB811637	AB811638	AB811639	AB811630	AB811631	AB811632	AB811633	AB811634
EHDV Isolate CC 304-06	HM641772	HM641773	HM641774	HM641775	HM641777	HM641700	HM641778	HM641776	HM641779	HM641781

**Table 3:** Amino acid percentage identities for Segment 2 (VP2) on the bottom left and Segment 7 (VP7) on the top right.

Virus	Abadina	Bunyip Creek	CSIRO Village	D’Aguilar	Kasba	Marrakai	Palyam	Petevo	Vellore	Apies River	Gweru	Marondera	Nyabira
Abadina		98.87	98.87	98.97	99.72	99.72	98.87	99.44	98.59	100.00	99.72	100.00	100.00
Bunyip Creek	39.26		100.00	100.00	98.87	98.87	98.87	99.15	97.75	98.87	98.59	98.87	98.87
CSIRO Village	51.95	37.86		100.00	98.87	98.87	98.87	99.15	97.75	98.87	98.59	98.87	98.87
D’Aguilar	38.40	47.95	36.70		98.87	98.87	98.87	99.15	97.75	98.87	98.59	98.87	98.97
Kasba	86.68	40.33	52.25	39.96		100.00	99.15	99.72	98.87	99.72	99.44	99.72	99.72
Marrakai	42.72	39.25	44.80	37.91	43.01		99.15	99.72	98.87	99.72	99.44	99.72	99.72
Palyam	37.63	46.78	35.85	95.82	38.80	36.83		99.44	98.03	98.87	98.59	98.87	98.87
Petevo	43.94	38.45	44.31	36.14	44.42	52.85	35.26		98.59	99.44	99.15	99.44	99.44
Vellore	85.19	39.65	51.07	39.57	98.11	42.33	38.41	43.84		98.59	98.31	98.59	98.59
Apies River	39.07	74.26	38.25	47.37	40.23	38.48	46.30	37.29	39.85		99.72	100.00	100.00
Gweru	51.95	37.86	99.90	36.70	52.25	44.80	35.85	44.31	51.07	38.25		99.72	99.72
Marondera	38.59	74.17	38.15	46.98	37.75	38.48	45.91	37.00	39.36	98.72	38.15		100.00
Nyabira	38.30	48.14	37.38	95.99	39.86	37.91	92.23	36.24	39.38	47.56	37.38	47.08	

## Supplementary Materials

The following are available online at https://www.mdpi.com/1999-4915/11/5/446/s1, Table S1: Models selected by jModel test for specified gene regions, Table S2: Amino acid percentage identities for Segment 1 (VP1) on the bottom left and Segment 3 (VP3) on the top right, Table S3: Amino acid percentage identities for Segment 4 (VP4) on the bottom left and Segment 6 (VP5) on the top right, Table S4: Amino acid percentage identities for Segment 9 (VP6) on the bottom left and Segment 5 (NS1) on the top right, Table S5: Amino acid percentage identities for Segment 8 (NS2) on the bottom left and Segment 10 (NS3) on the top right.
