# Supplementary material for: Phylogenetic Characterization of the Palyam Serogroup Orbiviruses
Source: Viruses. 2019 May 16;11(5):446. doi: 10.3390/v11050446 (PMC6563232; doi:10.3390/v11050446)
Supplement: Supplementary file 1 [file viruses-11-00446-s001.zip › Supplementary materials/Table S1.docx]

**Table S1.** Models selected by jModel test for specified gene regions

| Gene | Length  (bp/aa) | Model  (AIC) | Gamma | p-inv |
| --- | --- | --- | --- | --- |
| Segment 1 | 4070 | GTR + G | 0.1730 | NA |
| Segment 2 | 3564 | GTR + G | 0.7650 | NA |
| Segment 3 | 2802 | TIM2 + G | 0.1710 | NA |
| Segment 4 | 2044 | TIM2 + G | 0.2290 | NA |
| Segment 5 | 1941 | TIM2 + G | 0.3160 | NA |
| Segment 6 | 1667 | GTR + I + G | 0.5780 | 0.2510 |
| Segment 7 | 1212 | TVM + G | 0.2000 | NA |
| Segment 8 | 1272 | GTR + G | 0.2420 | NA |
| Segment 9 | 1334 | GTR + G | 0.2700 | NA |
| Segment 10 | 869 | TPM2uf + G | 0.2850 | NA |
