# Supplementary material for: Phylogenetic Characterization of the Palyam Serogroup Orbiviruses
Source: Viruses. 2019 May 16;11(5):446. doi: 10.3390/v11050446 (PMC6563232; doi:10.3390/v11050446)
Supplement: Supplementary file 1 [file viruses-11-00446-s001.zip › Supplementary materials/Table S2.docx]

**Table S2.** Amino acid percentage identities for Segment 1 (VP1) on the bottom left and Segment 3 (VP3) on the top right

| Virus | Kasba | Vellore | Abadina | CSIRO Village | Gweru | Marrakai | Petevo | Apies River | Marondera | Bumyip Creek | D’Aguilar | Palyam | Nyabira |
| --- | --- | --- | --- | --- | --- | --- | --- | --- | --- | --- | --- | --- | --- |
| Kasba |  | 99.45 | 98.24 | 98.57 | 98.35 | 98.90 | 97.58 | 98.24 | 98.35 | 98.46 | 98.35 | 97.25 | 98.35 |
| Vellore | 97.54 |  | 97.69 | 98.13 | 97.80 | 98.35 | 97.25 | 97.69 | 97.80 | 98.02 | 97.91 | 96.81 | 97.80 |
| Abadina | 94.32 | 94.40 |  | 97.69 | 99.89 | 98.02 | 98.13 | 99.67 | 99.89 | 97.58 | 97.47 | 97.69 | 99.89 |
| CSIRO Village | 96.85 | 97.39 | 93.32 |  | 97.80 | 98.57 | 96.92 | 97.91 | 97.80 | 99.89 | 99.78 | 97.25 | 97.80 |
| Gweru | 93.78 | 94.01 | 98.93 | 93.02 |  | 98.13 | 98.24 | 99.78 | 100.00 | 97.69 | 97.58 | 97.80 | 100.00 |
| Marrakai | 97.62 | 96.62 | 94.17 | 96.24 | 93.63 |  | 97.58 | 98.24 | 98.13 | 98.46 | 98.35 | 97.36 | 98.13 |
| Petevo | 94.40 | 94.47 | 94.09 | 94.09 | 93.48 | 94.55 |  | 98.02 | 99.24 | 96.92 | 96.81 | 98.13 | 98.24 |
| Apies River | 94.01 | 94.09 | 99.31 | 92.86 | 98.47 | 93.78 | 93.54 |  | 99.78 | 97.80 | 97.69 | 97.69 | 99.78 |
| Marondera | 93.63 | 93.71 | 98.70 | 92.86 | 99.23 | 93.40 | 93.32 | 98.23 |  | 97.69 | 97.58 | 97.80 | 100.00 |
| Bunyip Creek | 96.39 | 97.24 | 93.32 | 98.47 | 92.86 | 95.93 | 93.71 | 92.86 | 92.71 |  | 99.89 | 97.25 | 97.69 |
| D’Aguilar | 96.47 | 97.31 | 93.48 | 98.54 | 93.02 | 96.01 | 93.63 | 93.02 | 92.86 | 99.46 |  | 97.14 | 97.58 |
| Palyam | 93.40 | 93.55 | 93.55 | 93.71 | 93.40 | 94.01 | 95.70 | 93.17 | 93.25 | 93.40 | 93.32 |  | 97.80 |
| Nyabira | 94.01 | 94.09 | 99.08 | 93.32 | 99.54 | 93.86 | 93.71 | 98.62 | 99.31 | 93.17 | 93.32 | 93.48 |  |

**Table S3.** Amino acid percentage identities for Segment 7 (VP3) on the bottom left and Segment 3 (VP3) on the top right

| Virus | Abadina | Bunyip Creek | CSIRO Village | D’Aguilar | Kasba | Marrakai | Palyam | Petevo | Vellore | Apies River | Gweru | Marondera | Nyabira |
| --- | --- | --- | --- | --- | --- | --- | --- | --- | --- | --- | --- | --- | --- |
| Abadina |  | MH823477 | MH817078 | MH817097 | MH817117 | MH823377 | MH823397 | MH823417 | MH823437 | MH823457 |  |  |  |
| Bunyip Creek | 39.26 |  | MH817079 | MH817098 | MH817118 | MH823378 | MH823398 | MH823418 | MH823438 | MH823458 |  |  |  |
| CSIRO Village | 51.95 | 37.86 |  | MH817099 | MH817119 | MH823379 | MH823399 | MH823419 | MH823439 | MH823459 |  |  |  |
| D’Aguilar | 38.40 | 47.95 | 36.70 |  | MH817120 | MH823380 | MH823400 | MH823420 | MH823440 | MH823460 |  |  |  |
| Kasba | 86.68 | 40.33 | 52.25 | 39.96 |  | MH823381 | MH823401 | MH823421 | MH823441 | MH823461 |  |  |  |
| Marrakai | 42.72 | 39.25 | 44.80 | 37.91 | 43.01 |  | MH823402 | MH823422 | MH823442 | MH823462 |  |  |  |
| Palyam | 37.63 | 46.78 | 35.85 | 95.82 | 38.80 | 36.83 |  | MH823423 | MH823443 | MH823463 |  |  |  |
| Petevo | 43.94 | 38.45 | 44.31 | 36.14 | 44.42 | 52.85 | 35.26 |  | MH823444 | MH823464 |  |  |  |
| Vellore | 85.19 | 39.65 | 51.07 | 39.57 | 98.11 | 42.33 | 38.41 | 43.84 |  | MH823465 |  |  |  |
| Apies River | 39.07 | 74.26 | 38.25 | 47.37 | 40.23 | 38.48 | 46.30 | 37.29 | 39.85 |  |  |  |  |
| Gweru | 51.95 | 37.86 | 99.90 | 36.70 | 52.25 | 44.80 | 35.85 | 44.31 | 51.07 | 38.25 |  |  |  |
| Marondera | 38.59 | 74.17 | 38.15 | 46.98 | 37.75 | 38.48 | 45.91 | 37.00 | 39.36 | 98.72 | 38.15 |  |  |
| Nyabira | 38.30 | 48.14 | 37.38 | 95.99 | 39.86 | 37.91 | 92.23 | 36.24 | 39.38 | 47.56 | 37.38 | 47.08 |  |
