# Supplementary material for: Phylogenetic Characterization of the Palyam Serogroup Orbiviruses
Source: Viruses. 2019 May 16;11(5):446. doi: 10.3390/v11050446 (PMC6563232; doi:10.3390/v11050446)
Supplement: Supplementary file 1 [file viruses-11-00446-s001.zip › Supplementary materials/Table S3.docx]

**Table S3.** Amino acid percentage identities for Segment 4 (VP4) on the bottom left and Segment 6 (VP5) on the top right

| Virus | Kasba | Vellore | Abadina | CSIRO Village | Gweru | Marrakai | Petevo | Apies River | Marondera | Bumyip Creek | D’Aguilar | Palyam | Nyabira |
| --- | --- | --- | --- | --- | --- | --- | --- | --- | --- | --- | --- | --- | --- |
| Kasba |  | 100.00 | 97.72 | 93.55 | 97.91 | 98.48 | 84.44 | 78.37 | 78.37 | 79.70 | 78.94 | 78.56 | 78.4 |
| Vellore | 98.45 |  | 97.72 | 93.55 | 97.91 | 98.48 | 84.44 | 78.37 | 78.37 | 79.70 | 78.94 | 78.56 | 78.94 |
| Abadina | 93.79 | 93.32 |  | 94.50 | 99.05 | 97.34 | 84.44 | 78.75 | 78.75 | 79.89 | 78.94 | 78.56 | 78.94 |
| CSIRO Village | 95.19 | 96.43 | 92.70 |  | 95.07 | 93.55 | 81.02 | 77.80 | 77.80 | 79.32 | 77.61 | 81.97 | 77.61 |
| Gweru | 94.10 | 93.63 | 99.53 | 93.01 |  | 97.53 | 84.25 | 78.75 | 78.75 | 79.89 | 78.94 | 78.37 | 78.94 |
| Marrakai | 94.57 | 94.41 | 93.48 | 95.50 | 93.48 |  | 84.06 | 78.75 | 78.75 | 79.32 | 78.75 | 77.99 | 78.75 |
| Petevo | 93.79 | 93.32 | 94.25 | 92.08 | 94.25 | 93.32 |  | 78.37 | 78.37 | 78.37 | 80.08 | 77.99 | 79.70 |
| Apies River | 94.25 | 93.79 | 98.60 | 93.48 | 98.76 | 93.79 | 94.41 |  | 100.00 | 92.98 | 84.53 | 78.94 | 83.11 |
| Marondera | 93.79 | 93.32 | 98.76 | 92.70 | 98.91 | 93.32 | 94.25 | 98.91 |  | 92.98 | 84.63 | 78.94 | 83.11 |
| Bunyip Creek | 95.19 | 94.57 | 93.63 | 95.19 | 93.63 | 97.83 | 92.86 | 93.79 | 93.32 |  | 84.06 | 78.75 | 84.25 |
| D’Aguilar | 95.34 | 94.72 | 93.79 | 95.03 | 93.79 | 97.98 | 93.01 | 94.10 | 93.63 | 99.53 |  | 76.66 | 96.58 |
| Palyam | 94.88 | 94.25 | 93.94 | 93.17 | 93.94 | 94.57 | 94.72 | 94.25 | 93.94 | 94.25 | 94.41 |  | 77.23 |
| Nyabira | 94.10 | 93.63 | 99.22 | 93.01 | 99.38 | 93.79 | 94.57 | 99.07 | 99.22 | 93.94 | 94.10 | 94.10 |  |
