# Supplementary material for: Phylogenetic Characterization of the Palyam Serogroup Orbiviruses
Source: Viruses. 2019 May 16;11(5):446. doi: 10.3390/v11050446 (PMC6563232; doi:10.3390/v11050446)
Supplement: Supplementary file 1 [file viruses-11-00446-s001.zip › Supplementary materials/Table S4.docx]

**Table S4.** Amino acid percentage identities for Segment 9 (VP6) on the bottom left and Segment 5 (NS1) on the top right

| Virus | Kasba | Vellore | Abadina | CSIRO Village | Gweru | Marrakai | Petevo | Apies River | Marondera | Bumyip Creek | D’Aguilar | Palyam | Nyabira |
| --- | --- | --- | --- | --- | --- | --- | --- | --- | --- | --- | --- | --- | --- |
| Kasba |  | 99.82 | 96.52 | 95.50 | 95.40 | 95.14 | 94.06 | 96.76 | 96.76 | 95.32 | 95.32 | 93.17 | 96.76 |
| Vellore | 100.00 |  | 96.40 | 95.32 | 96.22 | 94.96 | 93.88 | 96.58 | 96.58 | 95.14 | 95.14 | 92.99 | 96.58 |
| Abadina | 87.40 | 87.46 |  | 96.04 | 99.46 | 95.86 | 94.60 | 99.82 | 99.82 | 96.04 | 96.04 | 94.06 | 99.82 |
| CSIRO Village | 94.62 | 94.62 | 86.38 |  | 95.86 | 98.20 | 93.71 | 96.22 | 96.22 | 98.2 | 99.10 | 92.27 | 96.22 |
| Gweru | 87.10 | 87.10 | 95.34 | 86.02 |  | 95.68 | 94.06 | 99.64 | 99.64 | 95.86 | 95.86 | 93.88 | 99.64 |
| Marrakai | 97.13 | 97.13 | 86.74 | 94.62 | 86.38 |  | 93.35 | 96.04 | 96.04 | 97.84 | 98.02 | 92.45 | 96.04 |
| Petevo | 90.32 | 90.32 | 88.53 | 89.61 | 89.25 | 90.32 |  | 94.42 | 94.42 | 93.71 | 93.71 | 93.71 | 94.24 |
| Apies River | 87.10 | 87.10 | 95.14 | 86.02 | 97.13 | 85.66 | 88.53 |  | 100.00 | 96.22 | 96.22 | 94.24 | 100.00 |
| Marondera | 86.38 | 86.38 | 94.62 | 85.30 | 99.28 | 85.66 | 88.53 | 96.42 |  | 96.22 | 96.22 | 94.24 | 100.00 |
| Bunyip Creek | 94.27 | 94.27 | 86.02 | 97.49 | 87.10 | 94.27 | 88.89 | 87.10 | 86.38 |  | 99.46 | 92.45 | 96.22 |
| D’Aguilar | 94.62 | 94.62 | 85.66 | 97.85 | 86.02 | 94.62 | 89.96 | 86.02 | 85.30 | 98.92 |  | 92.27 | 96.22 |
| Palyam | 89.96 | 89.96 | 88.53 | 88.89 | 87.81 | 90.68 | 92.47 | 87.10 | 87.10 | 88.17 | 88.53 |  | 94.24 |
| Nyabira | 87.46 | 87.46 | 95.70 | 86.38 | 96.08 | 86.02 | 88.89 | 96.06 | 95.34 | 86.74 | 86.38 | 88.89 |  |
