# Supplementary material for: Phylogenetic Characterization of the Palyam Serogroup Orbiviruses
Source: Viruses. 2019 May 16;11(5):446. doi: 10.3390/v11050446 (PMC6563232; doi:10.3390/v11050446)
Supplement: Supplementary file 1 [file viruses-11-00446-s001.zip › Supplementary materials/Table S5.docx]

**Table S5.** Amino acid percentage identities for Segment 8 (NS2) on the bottom left and Segment 10 (NS3) on the top right

| Virus | Kasba | Vellore | Abadina | CSIRO Village | Gweru | Marrakai | Petevo | Apies River | Marondera | Bumyip Creek | D’Aguilar | Palyam | Nyabira |
| --- | --- | --- | --- | --- | --- | --- | --- | --- | --- | --- | --- | --- | --- |
| Kasba |  | 96.79 | 94.04 | 92.20 | 94.04 | 94.95 | 96.33 | 93.12 | 93.58 | 92.66 | 93.12 | 92.20 | 94.04 |
| Vellore | 99.71 |  | 92.20 | 91.28 | 92.20 | 96.79 | 99.54 | 91.28 | 91.74 | 91.28 | 91.74 | 92.66 | 92.20 |
| Abadina | 92.04 | 92.33 |  | 93.58 | 100.00 | 93.12 | 92.66 | 99.08 | 99.54 | 94.04 | 94.50 | 94.04 | 100.00 |
| CSIRO Village | 89.68 | 89.97 | 91.15 |  | 93.58 | 93.12 | 91.74 | 93.58 | 93.12 | 98.62 | 99.08 | 94.95 | 93.58 |
| Gweru | 92.63 | 92.92 | 99.41 | 91.74 |  | 93.12 | 92.66 | 99.08 | 99.54 | 90.04 | 94.50 | 94.04 | 100.00 |
| Marrakai | 97.35 | 97.64 | 90.86 | 89.68 | 91.45 |  | 96.33 | 93.12 | 92.66 | 92.66 | 93.12 | 94.50 | 93.12 |
| Petevo | 92.04 | 92.33 | 92.04 | 92.92 | 92.63 | 91.15 |  | 91.74 | 92.20 | 91.74 | 92.20 | 93.12 | 92.66 |
| Apies River | 92.04 | 92.33 | 99.41 | 91.74 | 99.41 | 90.86 | 92.04 |  | 98.62 | 90.04 | 94.50 | 94.04 | 99.08 |
| Marondera | 92.33 | 92.63 | 99.11 | 91.45 | 99.70 | 91.15 | 92.33 | 99.11 |  | 93.58 | 94.04 | 93.58 | 99.54 |
| Bunyip Creek | 89.09 | 89.38 | 89.97 | 97.35 | 90.56 | 89.38 | 93.22 | 90.56 | 90.27 |  | 99.54 | 95.41 | 94.04 |
| D’Aguilar | 89.09 | 89.38 | 89.97 | 97.35 | 90.56 | 89.38 | 93.22 | 90.56 | 90.27 | 99.41 |  | 95.87 | 94.50 |
| Palyam | 95.28 | 94.99 | 89.38 | 89.38 | 89.97 | 93.22 | 93.22 | 89.38 | 89.68 | 89.38 | 89.38 |  | 94.04 |
| Nyabira | 92.63 | 92.92 | 99.41 | 91.74 | 100.00 | 91.45 | 92.63 | 99.41 | 99.41 | 90.56 | 90.56 | 89.97 |  |
